# Targeting Oxidative Stress Mechanisms to Treat Alzheimer's and Parkinson's Disease: A Critical Review

**DOI:** 10.1155/2022/7934442

**Published:** 2022-07-31

**Authors:** Abdullahi Tunde Aborode, Manas Pustake, Wireko Andrew Awuah, Mariam Alwerdani, Parth Shah, Rohan Yarlagadda, Shahzaib Ahmad, Inês F. Silva Correia, Ayush Chandra, Esther Patience Nansubuga, Toufik Abdul-Rahman, Aashna Mehta, Omar Ali, Shekinah Obinna Amaka, Yves Miel H. Zuñiga, Anastasiia D. Shkodina, Oko Christian Inya, Bairong Shen, Athanasios Alexiou

**Affiliations:** ^1^Department of Chemistry, Mississippi State University, Starkville, USA; ^2^Department of Internal Medicine, Grant Government Medical College and Sir JJ Group of Hospitals, Mumbai, India; ^3^Harvard Medical School, Harvard University, Boston, MA, USA; ^4^Sumy State University, Ukraine; ^5^Mansoura Faculty of Medicine, Mansoura University, Egypt; ^6^MGM Medical College and Hospital, Aurangabad, India; ^7^Rowan University School of Osteopathic Medicine, Stratford, New Jersey, USA; ^8^Mayo Hospital Lahore, Pakistan; ^9^School of Medicine, Faculty of Health, Education, Medicine & Social Care, Anglia Ruskin University, Bishop Hall Lane, Chelmsford CM1 1SQ, UK; ^10^Tianjin Medical University, Tianjin, China; ^11^Leeds Medical School, University of Leeds, UK; ^12^University of Debrecen-Faculty of Medicine, Debrecen, Hungary; ^13^Norwich Medical School, University of East Anglia, Norwich, UK; ^14^University of the Philippines Manila, Philippines; ^15^Poltava State Medical University, Poltava, Ukraine; ^16^Municipal Enterprise “1 City Clinical Hospital of Poltava City Council”, Poltava, Ukraine; ^17^Institute of Applied Health Research, University of Birmingham, Birmingham, UK; ^18^West China School of Nursing/Institutes for Systems Genetics, Frontiers Science Center for Disease-related Molecular Network, West China Hospital, Sichuan University, 610041 Chengdu, Sichuan, China; ^19^Novel Global Community Educational Foundation, Hebersham, 2770 NSW, Australia; ^20^AFNP Med Austria, 1010 Wien, Austria

## Abstract

Neurodegenerative disorders such as Alzheimer's disease (AD) and Parkinson's disease (PD) are becoming more frequent as the age increases. Contemporary therapies provide symptom resolution instead of targeting underlying pathological pathways. Consequently, there is considerable heterogeneity in response to treatment. Research has elucidated multiple potential of pathophysiological mechanisms contributing to neurodegenerative conditions, among which oxidative stress pathways appear to be suitable drug targets. The oxidative stress pathway has given rise to numerous novel pharmacological therapies that may provide a new avenue for neurodegenerative diseases. For example, SKQ (plastoquinone), MitoVitE, vitamin E, SOD mimic, MitoTEMPO (SOD mimetic), and bioactive molecules like curcumin and vitamin C have indeed been examined. To better understand how oxidative stress contributes to neurodegenerative diseases (such as Alzheimer's and Parkinson's), we analyzed the medicinal qualities of medicines that target markers in the cellular oxidative pathways. The specific pathway by which mitochondrial dysfunction causes neurodegeneration will require more investigation. An animal study should be carried out on medications that tackle cellular redox mechanisms but are not currently licensed for use in the management of neurodegenerative conditions.

## 1. Introduction

Since they are both neurological diseases, Alzheimer's disease (AD) and Parkinson's disease (PD) remain untreated [1]. PD affects approximately one percent of the overall of the individuals over the age of 60, while Alzheimer's disease (AD) affects approximately four percent of the overall of the people above the age of 65 [2, 3].

**Figure 1:**
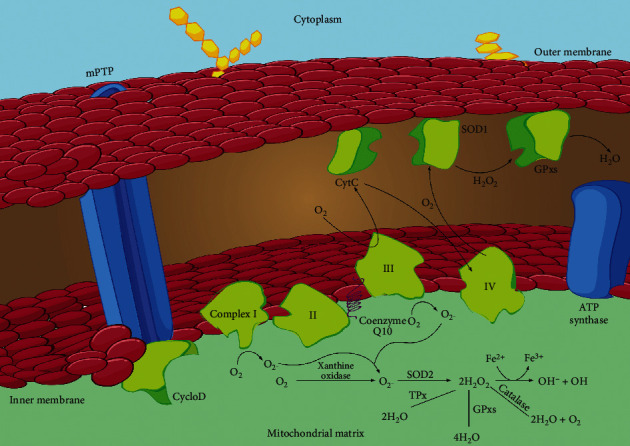
Defining the role of free radicals and antioxidants in cellular oxidative development.

Oxidative stress, which is a hallmark of aging, is the major trigger for both disorders. Mitochondrial dysfunction is the redox condition that results from an imbalance between the production and elimination of reactive oxygen species (ROS) (Figure 1). It is impossible to avoid ROS even though they metabolize all significant cellular components, especially DNA, RNA, protein, and triglycerides [4]. ROS have been used in cell signaling pathways when they are present in high concentrations.

Neuropathologically, PD is distinguished by

- a reduction in the amount of dopaminergic currently offered for neurotransmission in the substantia nigra pars compacta (SNpc) resulting in a loss of dopamine pathways; and
- there is a development of Lewy bodies, neurofibrillary tangles aggregates that incorporate microfibrils synuclein [5]

Disruption of circuitry that controls movement and posture is caused by deficit in the dopamine neurotransmitter in the SNpc, which results in symptoms including slowness of movement and relaxation trembling. Parkinson's disease is also known to cause nonmotor signs such as sleep disorders, anxiety, memory impairment, and malfunctions of the autonomous nervous system and the senses [6, 7]. The neurodegenerative disorders expression of AD is substantially more extensive, with functional decline occurring in conjunction with amyloid plaques, neurofibrillary tangles, and cerebral amyloid angiopathy [8]. The final outcome of degenerative progressions that have yet to be defined in AD and PD is oxidative stress as a result of cellular malfunction.

It is still not obvious how oxidative stress plays a role in either disease's onset or progression, but this is one theory that has been floated by some researchers. Previous studies have attempted to disseminate the role of oxidative stress in the pathophysiology of neurodegenerative diseases [9].

Treatments aiming cellular oxidative pathways may be beneficial in the management of neurodegenerative disorders, particularly those associated with endothelial dysfunction recently. Oxidative stress is a major contributor to neurodegeneration in general and to the development of both Parkinson's disease and Alzheimer's disease (AD). There will also be an examination of present and upcoming treatments for oxidative stress. For these new medicines, future outlooks are presented to discuss their possibility for disease-modifying.

## 2. The Role of Oxidative Stress in Neurodegeneration

### 2.1. Mechanisms of Oxidative Stress

**Figure 2:**
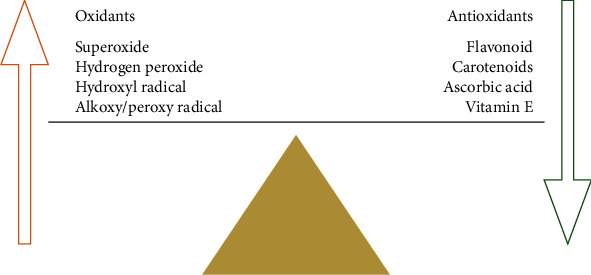
Reactive oxygen species (ROS) production in mitochondria (mtROS).

**Figure 3:**
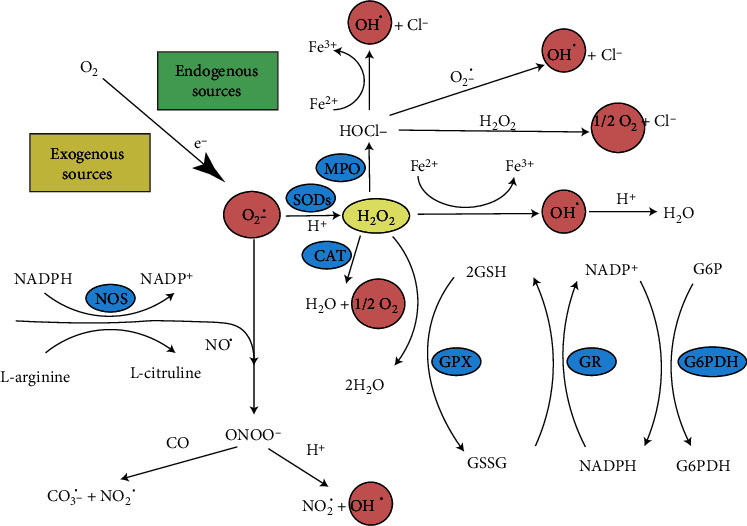
Radical species development.

The electrons in the outermost electron shells of reactive species (subatomic particles, molecules, or ions) are unpaired, and this gives them a high degree of responsiveness. Endogenous reactive oxygen species (also known as ROS) have the ability to metabolize macromolecules since they are capable of doing so. Numerous metabolic reactions, such as oxidative phosphorylation, generate ROS. Mitochondrial-derived reactive oxygen species (mtROS) (Figures 2 and 3) include singlet oxygen (O_2_), superoxide anion (O_2_^•−^), hydrogen peroxide (H_2_O_2_), nitric oxide (NO^•^), hydroxyl radical (OH^•^), and hydroxyl ion (OH^−^). Xanthine oxidase (XO) or mitochondrial respiratory chain complexes I (NADH dehydrogenase) and III (bc1 complex) first convert oxygen to superoxide anion [3, 10] (Figure 2). In both the matrix and the intermembranous region, complex III generates a superoxide anion (Figure 2) [11].

A patient's condition, age, and hormonal status all affect how much of these complexities are present in IMM. In order to produce hydrogen peroxide, the superoxide anion must first be converted by SOD. Hydrogen peroxide can be detoxicated to water and oxygen using glutathione peroxidase, catalase (CAT), or thioredoxin peroxidase (TPx) [12, 13]. It can also be converted to hydroxyl radicals and hydroxyl ions via the Fenton reaction (Figure 3) [14].

At physiological levels, ROS are sequestered by endogenous antioxidants such as glutathione peroxidase and superoxide dismutase. The central nervous system (CNS) is especially vulnerable to oxidative stress [15]. The rapid energy level of cerebral cortex, the greater amount of polyunsaturated fatty acids (PUFA) in the biological synapses, and the native autooxidative pathways engaged in neurotransmitters are all factors that contribute to this sensitivity.

Despite unsaturated fatty acids comprising 20% of the brain's total fatty acid content, only around 3% of the total glutathione peroxidase in the human liver is found in the brain. Superoxide dismutase levels in brain tissue are comparable to those found in the heart and liver. Iron, often found as iron-neuromelanin complexes in dopaminergic neurons, under pathological conditions may act as a cofactor for producing ROS [16]. All the above contribute to the greater susceptibility of CNS to ROS. Locally increased levels of ROS can precipitate mitochondrial dysfunction by damaging membrane proteins and may cause adverse mutations in mitochondrial DNA [17, 18].

The endpoint of this cascade is neuroinflammation and neuronal dysfunction as seen in PD and AD [19]. Currently biological studies posit that such consequences are primarily the result of mitochondrial dysfunction, secondary from oxidative stress, leading to neurodegeneration [20–22].

### 2.2. Reactive Species as Modulators of the Neurological Function

**Figure 4:**
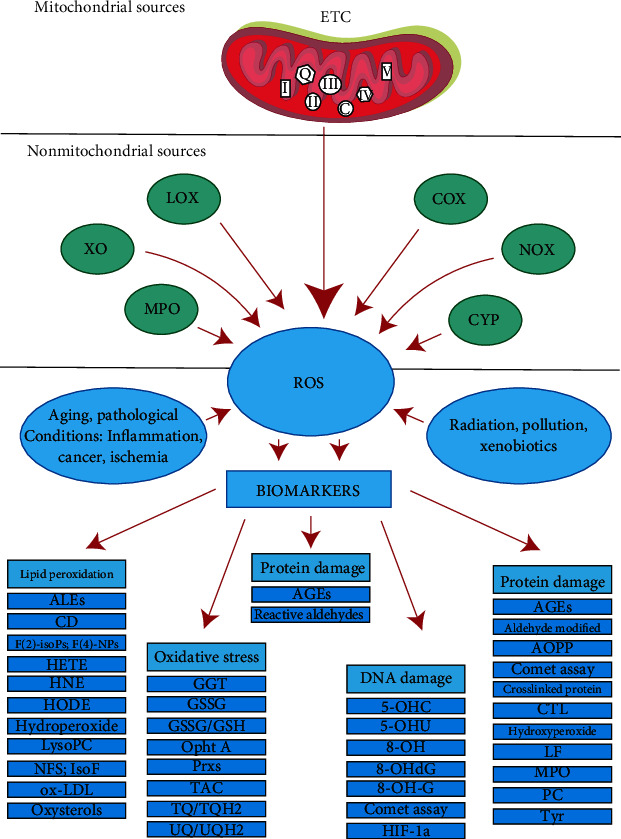
Cellular redox indicators' derivation [27].

Reactive oxygen species (ROS) modulate many aspects of neurological function by acting as secondary messengers in several pathways (Figure 4). There are two types of synaptic plasticity: LTP and LTD, which refer to an improvement in synaptic performance as well as a decrease in signal transduction [23].

ROS are relevant in the hippocampus and spinal cord where they partake in LTP [24]. Secondly, ROS activates microglia and astrocytes causing the ongoing release of proinflammatory cytokines and chemokines analogous to the systemic, low-grade inflammation that occurs secondary to malignancy. Thirdly, ROS play a crucial role in the differentiating of neurons by influencing the multiplication of brain progenitor and epigenetics.

Fourthly, ROS inhibits sodium currents needed for action potentials via the oxidation of thiol groups (Figure 4). Interestingly the opening of voltage-gated calcium channels is enhanced by ROS [25, 26].

Complex I: NADH dehydrogenase; II: succinate dehydrogenase; III: bc1 complex; IV: cytochrome C oxidase; V: ATP synthase; Q: Ubiquinone; Cyt C: cytochrome C; Cyclo D: Cyclophilin D; mPTP: mitochondrial permeability transition pore; SOD: superoxide dismutase; GPxs: glutathione peroxidase; TPx: thioredoxin peroxidase. See text below for details [27].

### 2.3. Oxidative Stress in the Pathogenesis of PD and AD

Postmortem frontal brain samples from people aged 26 to 126 years showed a decline in genes linked to synapse formation, vesicular transport, and mitochondrial activity beyond the age of 40. The response to stress and antioxidants and DNA fix genes were then upregulated as a result of these modifications [28].

PD patients' nigral neurons, which are particularly vulnerable to mitochondrial malfunction due to high levels of oxidative metabolism, have been the subject of extensive investigation since the 1980s [2]. Regarding AD, though its pathophysiology has been explained through the amyloid and the neurofibrillary tangles, research has also implicated other mechanisms in its development and progression; among these mechanisms is mitochondrial dysfunction and oxidative stress [2], though, most authors agree that mitochondrial dysfunction precedes amyloid plaque deposition and is thus not the underlying cause.

In postmortem tissues, elevated levels of ATP were found in patients with AD both in cerebral structures and in peripheral tissues, signifying the presence of mitochondrial dysfunction. Complex I, III, and IV inadequacies have been observed in postmortem dissections thus far. Apart from mitochondrial dysfunction changes, there have also been changes in morphology and distribution of mitochondria, with research describing the length reduction and increase in numbers [2].

### 2.4. Novel Therapies Targeting Oxidative Stress Pathways

The capacity of these medications to pass the blood-brain barrier (BBB) is a critical hurdle when developing innovative therapeutics for neurodegenerative illness. According to research, the disease associated with complex 1 is the major source of mitochondrial dysfunction [29]; treatments that address this are being explored, including substances like SKQ (plastoquinone), MitoVitE (vitamin E), MitoTEMPO (SOD mimic), MCAT (catalase), MitoPBN (CoQ), and phenyl tert butylnitrone conjugation, as well as other chemicals.

Others are lipophilic cation-based tetrapeptide compounds or choline esters of glutathione and N-acetyl l-cysteine that can penetrate cells. There has been evidence that MitoQ slows down the onset of Alzheimer's disease by decreasing A-induced neurodegeneration in neuronal cells, reducing free radical generation [30, 31].

Intracellular enzymes such as superoxide dismutase and glutathione peroxidase provide some protection from ROS. Synthetic molecules such as butylated hydroxytoluene, butylated hydroxyanisole, and ethoxyquin PAPLAL (mixture of Pd and Pt NPs) have been designed to mimic these enzymes but may produce adverse effects resulting from systemic administration [31].

An erythropoietin (Neuro-EPO) intranasally administered shields from inflammation and neurological assaults, restoring impairments in recollection, acquisition, and recognition of new images while also reducing antioxidant activity [32, 33].

Due to the Nrf2–NF-B signaling axis, Cyclo (His-Pro) seems to have some anti-inflammatory and stress activities, as well as ability to repair neuronal functionality Antioxidant defenses can be activated and apoptosis inducing and inflammation response can be reduced by increasing the nuclear level of Nrf2 and preventing IB degradation [34].

Studies suggest Glial activation-mediated inflammation has some role in Parkinson's disease (PD), via the Glia maturation factor (GMF) [35]. Modified GMF articulation directly influences the creation of reactive oxygen species by 1-methyl-4-phenylpyridinium (MPP +). GMF suppression correlated with a decline in reactive oxygen species and resulted in downregulation of NF-kappaB-induced creation of TNFalpha and IL-1b. Thus, it decreased lipid peroxidation levels and increased levels of glutathione [35].

Metal-protein attenuating compounds (MPACs) interrupt the abnormal metal-protein interaction and normalize its distribution by competing with target proteins for metal ions [36].

Autopsies performed on brain tissue of PD and AD patients show involvement of transition metals during the formation of cytotoxic tissue aggregates, and metal chelating therapy has demonstrated significant efficacy in certain PD models by preventing lipid peroxidation, although long term use might interfere with normal physiological function [37]. Although these compounds show great results, they are still at the experimental phase, and their clinical application is being investigated.

### 2.5. Oxidative Stress Drug Targets in Anti-Alzheimer's Disease Therapy

Antioxidant properties of medications used to treat Alzheimer's disease show considerable differences based on dose and AD model. Tacrine, which was the first anticholinesterase inhibitor approved by the Food and Drug Administration (FDA), showed reduction in overall survival (OS) in an animal Alzheimer's disease model [38] and at a dose of 50–800 g/kg i.m. increased FRAP, and hence “antioxidant efficacy” [39], without raising any sign of OS-associated damage in brain tissue. Tacrine has a favorable effect when provided in doses that improve the antioxidant system without increasing oxidative stress-induced damage in brain tissue [40].

Donepezil, another cholinesterase inhibitor used in Alzheimer's disease patients, resulted in dose-dependent effects on antioxidant capacity and reduced lipid peroxidation when administered in mice AD models at modest doses. In the APPswe/PS1 transgenic mice AD model, donepezil did not decrease OS biomarkers or show significant antioxidant activity [41]. Hence, discrepancy in results from different studies suggests that adaptations to its use may be the source of the observed discrepancy in outcomes in transgenic and nontransgenic AD mice models.

Rivastigmine, another drug used to treat of Alzheimer's disease, does not show to decrease lipid peroxidation or replenish GSH in an AD rat brain model [42], despite a previous report indicating antioxidant capabilities for rivastigmine when Alzheimer's disease was produced in rats by aluminum chloride treatment [43].

A single study found that another AChE inhibitor, galantamine, might lower OS, leading to decreased lipid peroxidation, nitrate, and GSSG levels, as well as increased SOD activity and lower GSH levels, while also restoring cognitive impairments [44].

Studies performed on Alzheimer's disease preclinical models demonstrate Memantine to decrease oxidative-stress induced damage to cortex and hippocampus proteins, improving age-related recognition memory in senior rats [45]. Memantine also reduced the frequency of inducible forms of NOS in an A25–35 AD model [46] and, in addition, ROS and nitrate levels in the hippocampus and cortex in a streptozotocin AD model [47] as well as a kainic acid-induced model of dementia [47]. However, this effect was not observed in the striatum [45].

Immunomodulatory agents such as Fingolimod or FTY720, Tanshinone I, Lenalidomide, Thalidomide, Ginsenoside Rg1, CNI-1493, Pycnogenol, and C5aR antagonist DF3016A demonstrate anti-inflammatory action, thereby reducing OS and lipid peroxidation products and decreasing microglial, astrocytic, and T cell activities. They might be used as a preventative measure, and there is some evidence that they help with motor impairments and nigral dopaminergic neurotoxicity [48].

Over time, innovations in the field of nanomedicine have garnered widespread interest in the scientific community. Some compounds of interest are cerium oxide NPs, ceria/polyoxometalates hybrid, manganese tetroxide and manganese ferrite nanoparticles, yttrium oxide nanoparticles, iron oxide nanoparticles, copper nanoparticle clusters, cobalt oxide (Co3O4 NPs), and cobalt ferrite nanoparticles (CoFe2O4 NPs). Some compounds have antioxidant properties and other compounds like ceria mimic enzymes [49]. Nanoceria mimics SOD and CAT activity. Due to the mixed valency state of cerium oxide, it reacts with free radicals and detoxifies ROS and therefore may be neuroprotective.

Apart from metal and metal oxide nanoparticles described above, inorganic nanoparticles such as mesoporous silica nanoparticles (MSNs) can have potential applications as explained by their large surface area, structural tunability, and easy functionalization [50]. Therapeutic trials of these drugs and therapies in animal and human models are discussed below [38, 41, 42, 51–65]. In patients: (i) vitamin E (*α*-tocopherol, 800 IU/day) + vitamin C (500 mg/day) + *α*-lipoic acid (900 mg/day) ↓F2-isoprostane in CSF [51]; (ii) Coenzyme Q10 (400 mg ×3 times/day) NO CHANGE F2-isoprostane in CSF [51]; (iii) *ω*-3 (3 g/day contained 675 mg DHA and 975 mg EPA) NO CHANGE F2-isoprostane in urine, NO CHANGE PC in plasma [52]; (iv) *ω*-3 + *α*-lipoic acid (*ω*-3, 3 g/day contained 675 mg DHA and 975 mg EPA + *α*-lipoic acid, 600 mg/day in one tablet) NO CHANGE F2-isoprostane in urine NO CHANGE PC in plasma [52]; (v) vitamin C (1,000 mg/day) + vitamin E (400 IU/day) ↓oxidation of CSF [53]; (vi) Curcumin (1 or 4 g/day) NO CHANGE F2-isoprostane in plasma [54]; (vii) curcuminoids (2 or 4 g/day) NO CHANGE F2-isoprostane in CSF [55]. In animal models: (i) Schisantherin A 0.1 mg/kg for 5 days i.c.v., injection started after 3 days from A*β*1–42 injection (↓MDA in cerebral CTX, ↑SOD, ↑GPx, ↑GSH in HIP and cerebral CTX) [38]; (ii) vitamin E 150 mg/kg, p.o. for 27 days, administration began 7 days before A*β*1–42 i.c.v. (↓MDA, ↓PC, ↓Mn-SOD, ↓Zn, Cu-SOD, ↑GPx, Ø GR in cerebral CTX and HIP) [57]; (iii) Piperine 5 or 10 mg/kg p.o. 2 weeks before and 1 week after AF64A (↓MDA in HIP) [58]; (iv) S-allyl cysteine 30 mg/kg i.p. for 15 days pretreatment before streptozocin (↓TBARS, ↑GSH, ↑GPx, ↑GR in HIP) [59]; (v) Imperatorin 2×/day for 7 days with scopolamine injection (↓MDA, ↑SOD in CTX and HIP, ↑GPx in CTX and HIP, ↑GR in CTX) [42]; (vi) *α*-lipoic acid 30 mg/kg/day enriched diet for 10 months (↓HNE, Ø 3-NT in brain homogenates) [60]; (vii) vitamin C 125 mg/kg i.p. for 12 days (no change MDA in HIP) [61]; (viii) vitamin C low diet content 0.099 g/L of drinking water (↑MDA in CTX) [62]; (ix) melatonin 5 mg/kg p.o. for 5.5 months (↓MDA, ↓PC in HIP) [63]; (x) melatonin 10 mg/kg/day for 4 months intragastrically (↓TBARS, ↑GSH, ↑SOD in the brain homogenate) [64]

### 2.6. Oxidative Stress Drug Targets in Anti-Parkinson's Disease Therapy

Drugs like valproic acid, melatonin, ceftriaxone, and N-acetylcysteine have been shown in research to have oxidative impacts on human health. Antioxidative defense enzyme activity in mice was recovered after ceftriaxone treatment (glutathione, catalase, and SOD). Prior to taking ceftriaxone, Ropinirole considerably increased the protective effects [66].

Using 6-OHDA-induced SNpc dopaminergic neuronal damage in rat models, serofendic acid plays a protective role against 6-OHDA-induced oxidative stress parameters, such as 3-nitrotyrosine and 4-hydroxy-2-nonenal (4-HNE). When N-acetylcysteine was administered to animals, it induced an upsurge in the efficiency of lipid peroxylase, superoxide dismutase (SOD), and g-glutamyl transpeptidase (g-GTP) and a big decline in glutathione (GSH) levels and glutathione peroxidase function in the SNpc [67].

Ropinirole, a dopaminergic stimulant, increased GSH levels and CAT action, according to a new analysis. These PLGA nanoparticles (NPs) were developed to enhance the drug's effectiveness and distribution [55].

Parkinson's disease may be treated with the nootropic centrophenoxine (CPH). Catalase and superoxide dismutase (SOD) upregulated, whereas nitric oxide (NO) and citrulline levels decreased, according to one study [68]. These compounds seem to be promising; further studies are required to understand their efficacy in a natural setting because in the above studies, to produce selective DAergic neuronal degeneration in PD, rotenone-induced neurotoxicity was used as a preclinical model PD in mice. Many therapeutic trials of these drugs and therapies in animal and human models are conducted until date [66, 69–71]. These included (i) L-DOPA (200 mg/kg i.p. 2 injections/day for 4 weeks, coadministration with MPTP (no change GSH in SN) [69], (ii) Ropinirole 1, 5, or 3 mg/kg i.p. for 14 days, after MPTP (↑GSH, ↑CAT, ↓nitrate (only 1.5 mg/kg) in STR and CTX) [66]; (iii) Pramipexole 1 mg/kg i.p. 2 injections/day for 4 weeks, coadministration with MPTP (↑GSH in SN) [69]; and (iv) Deferoxamine 50 mg/kg p.o. for 14 days, coadministration with 6-OHDA (↓PC, ↑GSH, and ↑SOD in STR) [71].

## 3. Conclusions and Future Perspectives

Fundamentally, it is necessary to do extensive study on the inclusionary practices of ROS in the neurodegenerative process and AD, in order to analyze therapeutic targets that are noteworthy. Current medications, though effective, should not be considered optimal management of neurodegenerative disease. The drugs discussed above should undergo further evaluation for feasibly in human subjects. The effectiveness of pharmacological drugs that target the cellular oxidative system in reducing neurodegenerative should next be thoroughly tested in medical tests. Despite the fact that mitochondrial dysfunction is an important role in the development of AD and PD, little is known about the extent of this impact, which makes it difficult to put hypothetical pharmacological targets into reality in the real world.
